# Preptin Deficiency Does Not Protect against High‐Fat Diet‐Induced Metabolic Dysfunction or Bone Loss in Mice

**DOI:** 10.1002/jbm4.10777

**Published:** 2023-06-18

**Authors:** Emma J. Buckels, Joey Tan, Huai‐Ling Hsu, Yuting Zhu, Christina M. Buchanan, Brya G. Matthews, Kate L. Lee

**Affiliations:** ^1^ Department of Molecular Medicine and Pathology University of Auckland Auckland New Zealand; ^2^ Maurice Wilkins Centre for Molecular Biodiscovery University of Auckland Auckland New Zealand; ^3^ Department of Engineering Science University of Auckland Auckland New Zealand

**Keywords:** DIET, GLUCOSE TOLERANCE, INSULIN‐LIKE GROWTH FACTOR‐II, METABOLIC DYSFUNCTION, SECRETAGOGUE, SEXUAL DIMORPHISM, TRABECULAR BONE

## Abstract

Preptin is derived from the cleavage of the E‐peptide of pro‐insulin‐like growth factor (IGF)‐II and is an insulin secretagogue. Observational studies have linked elevated circulating preptin to metabolic dysfunction in humans; however, a causal role for preptin in metabolic dysfunction has not been established. Additionally, preptin can promote osteoblast proliferation and differentiation, suggesting a link with skeletal health. We previously described a global preptin knockout (KO) model. In this study, we sought to uncover the impact of preptin KO in mice on the response to a moderately high‐fat diet (HFD) and low‐fat diet (LFD). HFD groups had higher weight and fat mass gain, lower trabecular and cortical bone volume and fracture load, and higher liver triglycerides. In males, preptin deficiency led to lower blood glucose than wild‐type (WT) mice under LFD conditions. This was accompanied by differences in bone microarchitecture, including lower trabecular bone volume fraction, trabecular number, and lower cortical thickness. These differences were absent in female mice, although KO females had a HFD‐driven increase in fat mass and liver triglycerides that was absent in WT mice. Female WT mice had increased glucose‐stimulated insulin secretion under HFD conditions that was absent in female KO mice. Overall, preptin may have a detrimental impact on metabolism and a positive impact on bone health in male mice and may protect against liver fat storage in females while enabling islet compensation under HFD conditions. When we consider that serum preptin levels are elevated in humans of both sexes in pathological states in which insulin levels are elevated, the impact of preptin on comorbidity risk needs to be better understood. © 2023 The Authors. *JBMR Plus* published by Wiley Periodicals LLC on behalf of American Society for Bone and Mineral Research.

## Graphical abstract

Preptin is a pro‐insulin‐like growth factor (IGF)‐II peptide found in islet beta cells that has been shown to impact bone health and islet function. In male mice, preptin knockout (KO) led to lower blood glucose and lower bone density than wild‐type (WT) mice under low‐fat diet (LFD) conditions. These differences were absent in female mice, although KO females had a high‐fat diet (HFD)‐driven increase in fat mass and liver triglycerides that was absent in WT mice. Female WT mice had increased glucose‐stimulated insulin secretion under HFD conditions that was absent in female KO mice, revealing sexually dimorphic patterns of negative and positive impacts of preptin on metabolic health.

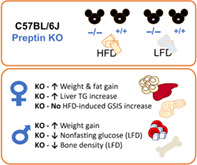

## Introduction

Type 2 diabetes mellitus (T2DM) is characterized by chronically elevated circulating glucose concentrations and develops when insulin secretion is insufficient to overcome insulin resistance in peripheral tissues.^(^ ^1^ ^)^ The ability of the pancreatic β‐cell to respond to circulating blood glucose concentrations by secreting an appropriate amount of insulin is critical for maintaining whole‐body metabolic homeostasis.^(^ ^2^, ^3^ ^)^ The canonical glucose‐stimulated insulin secretion (GSIS) pathway is well understood; however, many other secretagogues can modulate insulin secretion.^(^ ^4^, ^5^, ^6^ ^)^ Insulin granules contain numerous other hormones and proteins secreted alongside insulin including insulin‐like growth factor (IGF)‐II‐derived peptide hormones.^(^ ^7^, ^8^, ^9^ ^)^ One such hormone is preptin, a 34‐amino‐acid peptide derived from the cleavage of the E‐peptide of pro‐IGF‐II.^(^ ^10^ ^)^ Synthetic preptin increases GSIS in rodents in vivo and ex vivo,^(^ ^10^, ^11^ ^)^ suggesting that endogenous preptin has an autocrine role in β‐cell function. In humans, preptin levels appear to increase with increased circulating insulin or impaired glucose tolerance, such as in obesity, type 1 and T2DM, gestational diabetes mellitus, and polycystic ovarian syndrome.^(^ ^12^, ^13^, ^14^, ^15^, ^16^, ^17^, ^18^, ^19^, ^20^ ^)^ Aside from the associations identified in these observational studies, little is understood about the effects of endogenous preptin on whole‐body glucose metabolism. Nevertheless, due to its ability to increase insulin secretion,^(^ ^10^, ^11^ ^)^ preptin has been proposed as a potential therapeutic for T2DM.^(^ ^21^, ^22^ ^)^

Individuals with metabolic dysfunction, such as that seen in T2DM, have an increased risk of experiencing a fracture compared to nondiabetic subjects.^(^ ^23^, ^24^ ^)^ This increase is independent of fracture risk factors, including age, weight and height, and frequency of falls.^(^ ^25^, ^26^ ^)^ In contrast to nondiabetic individuals, there is no relationship between bone mineral density (BMD) and fracture incidence in individuals with T2DM.^(^ ^27^ ^)^ Therefore, although individuals with T2DM have increased BMD, the microstructure of bones is likely negatively affected by the disease.^(^ ^28^ ^)^ Hormones that regulate whole‐body glucose homeostasis also influence bone homeostasis. Insulin promotes bone formation while inhibiting bone resorption via direct effects on osteoblasts and osteoclasts.^(^ ^29^ ^)^ Interestingly, mouse studies indicate that hyperinsulinemia and insulin resistance in the absence of hyperglycemia, as is often seen in the early stages of T2DM,^(^ ^30^ ^)^ are associated with reduced bone turnover; this may ultimately lead to poorer bone quality and increased fragility.^(^ ^29^ ^)^ Moreover, preptin directly affects bone cells, promoting the proliferation and differentiation of osteoblasts in vitro and driving local bone formation in vivo.^(^ ^31^, ^32^, ^33^ ^)^ The role of endogenous preptin on bone mass and strength is unclear, particularly in metabolic dysfunction.

To address the dearth of information on the physiological function of endogenous preptin, we recently generated and characterized a novel mouse model with global genetic ablation of the preptin coding portion of the *Igf2* gene C57BL/6J‐Igf2tnEx3,4, hereafter referred to as preptin knockout (KO).^(^ ^34^ ^)^ Under basal conditions, bodyweight, body composition, β‐cell area, and fasted glucose concentrations were similar in preptin KO mice compared to wild‐type (WT) male and female mice up to 47 weeks of age. However, female KO mice had a diminished ability to mount an insulin response following glucose stimulation in vivo, which was not observed in male KO mice. This study demonstrated that while preptin is not critical for GSIS, endogenous preptin positively influences β‐cell function in female mice.

In this study, we fed preptin KO and WT littermate mice a high‐fat diet (HFD) for 14 weeks. In parallel, we compared KO and WT mice fed a low‐fat diet (LFD). HFD is a well‐characterized model of metabolic dysfunction in mice, inducing obesity, hyperinsulinemia, and impaired glucose tolerance.^(^ ^35^, ^36^ ^)^ Here we utilized a modest 46% fat diet to induce metabolic dysfunction without inducing full‐blown diabetes. The goals of this study were to determine whether preptin KO mice showed a different response than WT littermates to HFD with respect to body composition, glucose homeostasis, and bone microarchitecture and biomechanics. We hypothesized that KO mice would demonstrate more advanced metabolic dysfunction and decreased bone strength compared to WT mice.

## Methods

### Experimental animals

The Animal Ethics Committee at the University of Auckland approved all procedures (approval number 001984). Animals were bred and housed in the Vernon Jansen Unit under standard conditions (22°C, humidity at 40–45%, 12 h light: 12 h dark). Global preptin KO mice in a C57BL/6J background were generated as described previously.^(^ ^34^ ^)^ In this mouse, exons 3 and 4 of *Igf2* were truncated to remove the preptin sequence and intervening intron. All experimental mice were generated using heterozygous breeding pairs with maternal inheritance of the preptin KO allele. Mouse genotypes were confirmed by PCR, as described previously.^(^ ^34^ ^)^ Littermate pairs were used to decrease litter‐specific effects.

### Study design

**Fig. 1:**
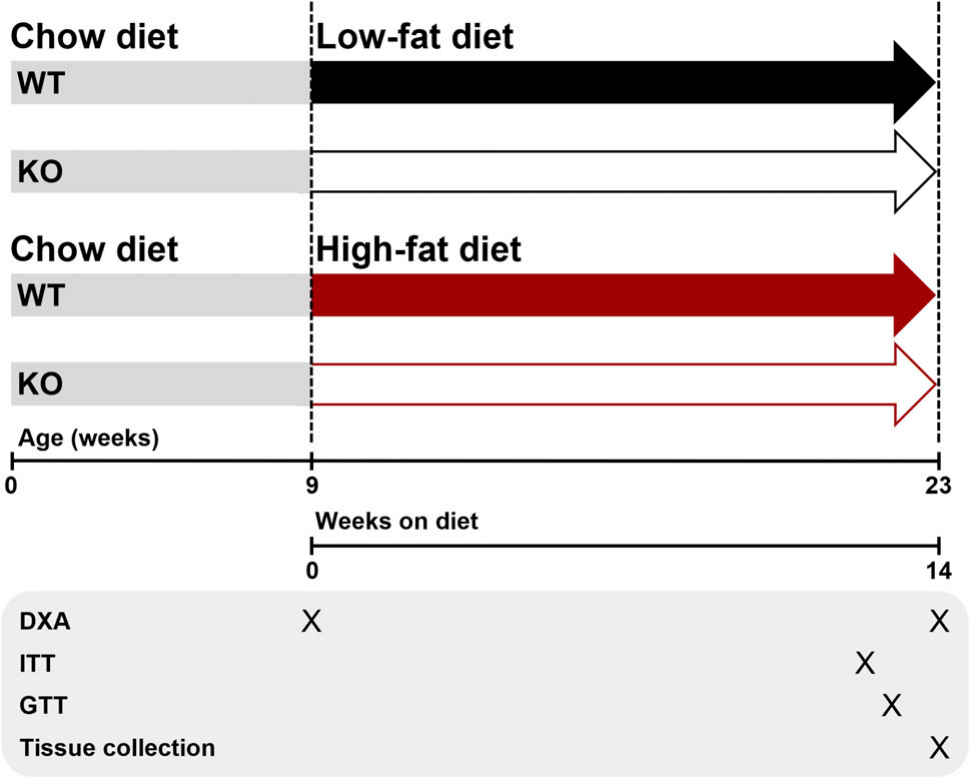
Study design. Both males and females were included in the study and analyzed separately, *n* = 11–15 per group.

Female and male mice were recruited at 9 ± 0.5 weeks of age and studied over 14 weeks (Fig. 1). Before recruitment for the study, mice were fed a standard chow diet (Envigo 2018; Teklad global 18% protein rodent diet). Littermate pairs were assigned to either a LFD containing 14.0% kcal from fat (SF16‐074; Specialty Feeds, Australia) or a HFD containing 46.0% kcal from fat (SF04‐027; Specialty Feeds). See Table S1 for full dietary composition.

### Glucose homeostasis

Nonfasting blood glucose concentrations from whole‐blood samples collected in the morning from the tail tip were measured weekly using an Accu‐Chek Performa portable blood glucose meter (Roche, Basel, Switzerland). Nonfasted blood glucose concentrations between 0 and 2 weeks and 10–12 weeks after dietary intervention were averaged for each mouse and included in data analyses. We chose 12 weeks as a cut‐off for this measure to avoid confounding these data with any potential metabolic stress experienced during the metabolic challenges performed in weeks 12–14.^(^ ^37^ ^)^ Following 12 weeks of dietary intervention, mice underwent an intraperitoneal insulin tolerance test (ITT). After 4 h fasting, a baseline blood glucose measurement was performed from the tail tip, then mice received insulin (0.5 mU/g Actrapid insulin) via intraperitoneal injection. Blood glucose measurements were taken at 15, 30, 60, 90, and 120 min after receiving a bolus. An oral glucose tolerance test (GTT) was performed after 13 weeks of dietary intervention. After 6 h fasting, mice received a glucose bolus (2 mg/g bodyweight) via oral gavage, and blood glucose measurements were performed as described for the ITT. Additional tail blood samples were collected at baseline and 30 min for measurement of serum insulin using the low‐sample volume protocol of a Mercodia mouse insulin ELISA kit (Mercodia, Uppsala, Sweden) for all samples with sufficient volume. Serum insulin readings for six fasting insulin samples were below the standard curve and were given a value of 0.006, which was below the lowest calculated level of 0.0072. Fasting (6 h) glucose (mmol/L) and fasting insulin (mU/ml) from the start of the GTT was used to calculate homeostasis model assessment of insulin resistance (HOMA‐IR): (Fasting glucose × fasting insulin)/(22.5), and homeostasis model assessment of beta‐cell function (HOMA‐β): (20× fasting insulin)/(Fasting glucose‐3.5).

### DXA scan

Body composition and BMD were measured by dual‐energy X‐ray absorptiometry (DXA; Lunar PIXImus Densitometer; GE Medical Systems Lunar., IL, USA) at baseline and following 14 weeks of dietary intervention. The PIXImus was unavailable for baseline and endpoint body composition scans for 10 male mice. These mice were excluded from the body composition analysis only. Mice were anesthetized using isoflurane inhalation throughout the scan. Analysis of each scan was performed with the Lunar PIXImus version 2.10 software by a single user.

### Tissue collection

Mice were euthanized by CO_2_ inhalation after 14 weeks of dietary intervention. Cardiac puncture was performed immediately following death, and serum was stored at −80°C. A portion of the liver was snap‐frozen in liquid nitrogen and stored at −80°C. Both hind limbs were harvested and stripped of all soft tissues. The right femur was stored in 4% paraformaldehyde for 48–72 h, then transferred to 70% ethanol and stored at 4°C. The left femur was wrapped in phosphate–buffered saline‐soaked gauze and stored at −20°C.

### Lipid metabolism

Nonesterified fatty acid (NEFA; NEFA C kit; Wako Chemicals, Osaka, Japan) and triglyceride (Triglyceride Assay Kit; PT, Pointe Scientific Inc., Canton, MI, USA) concentrations were measured in serum samples according to the manufacturers' instructions. Hepatic triglyceride content was measured by homogenizing approximately 25 mg of the snap‐frozen liver in 400 μL of Tris–HCl buffer (25 mM Tris–HCl, 0.5% Triton X‐100, 1 mM EDTA, 2 mM KCl, 50 mM MgCl, 5 mM NaV, pH 7.8). Triglyceride concentrations were then measured using a TRIGL kit according to the manufacturer's instructions (Roche, Switzerland). Triglyceride content was normalized to total protein concentration, measured using a Bicinchoninic acid (BCA) assay (Pierce, Copenhagen, Denmark).

### Micro–CT

The distal end of each femur was scanned using a Skyscan 1172 micro–CT (MCT) scanner (Bruker, Belgium), as previously described.^(^ ^38^ ^)^ The X‐ray voltage was 59 kV, the amperage was 167 μA, and a 0.5‐mm aluminum filter was used. Images were acquired with an isotropic voxel size of 7 μm, 180° of rotation, and a rotation step of 0.3°. After standardized reconstruction using NRecon software (Bruker, version 1.6.9.18), the data sets were analyzed using CTAn software (Bruker, version 1.18.8.0). Standardized parameters of trabecular and cortical bone microstructure were measured.^(^ ^39^ ^)^ The trabecular region of interest was 1.06 mm proximal to the growth plate and extended 1.41 and 2.11 mm in the proximal direction in females and males, respectively. The cortical region of interest was 5.99 mm proximal to the growth plate and extended 0.70 mm in the proximal direction in both sexes.

### Biomechanical testing

Left femora were loaded to failure in three‐point bending using an electromechanical testing system (Instron Universal Testing Machine, Model 5866, Instron, MA, USA, running Bluehill Universal software) with a 100‐N load cell.^(^ ^40^ ^)^ Samples were thawed at 4°C overnight in PBS and allowed to equilibrate at room temperature prior to testing. Soft tissue was removed, and femurs were placed on a custom‐made sample holder with 10 mm separation between the end supports. Femurs were oriented to make contact with the loading roller of the load cell at a consistent point along the midshaft. Specimens were tested at a compressive displacement rate of 1 mm/min until failure. Load–displacement data were measured in 0.1‐s intervals. Load–displacement data were used to calculate maximum load and stiffness, as previously described.^(^ ^41^ ^)^

### Statistical analysis

Mice were excluded from analyses if they developed a welfare issue during the experiment, which led to the exclusion of three animals in total (two due to malocclusion, and one had a tail injury). The group numbers were as follows: LFD female WT, *n* = 11; LFD female KO, *n* = 11; HFD female WT, *n* = 11; HFD female KO, *n* = 11; LFD male WT, *n* = 15; LFD male KO, *n* = 14: HFD male WT, *n* = 12; HFD male KO, *n* = 12. Data were analyzed using GraphPad Prism (version 9.0.2; San Diego, CA, USA) or SigmaPlot for Windows (version 14.0; Sysat Software Inc., San Jose, CA, USA), which conducted two‐way ANOVA comparisons. Significant outliers were identified using a ROUT (Q = 1%) analysis. Data were compared separately for each sex and were checked for normality using a Shapiro–Wilk test and log‐transformed when necessary. Data were compared using a two‐way ANOVA with genotype and diet as factors. Post hoc testing was applied when appropriate for two‐way ANOVA. Regression analysis was performed using linear modeling in R. Linear modeling was performed to assess the relationship between genotype and weight, body composition, and blood glucose, adjusting for sex and diet as indicated using the lm() function in the R package.^(^ ^42^ ^)^ Area under the curve (AUC) was calculated as the area above baseline for GTT and the area below baseline (negative area) for ITT.

## Results

### Bodyweight and fat mass increase in response to HFD

**Fig. 2:**
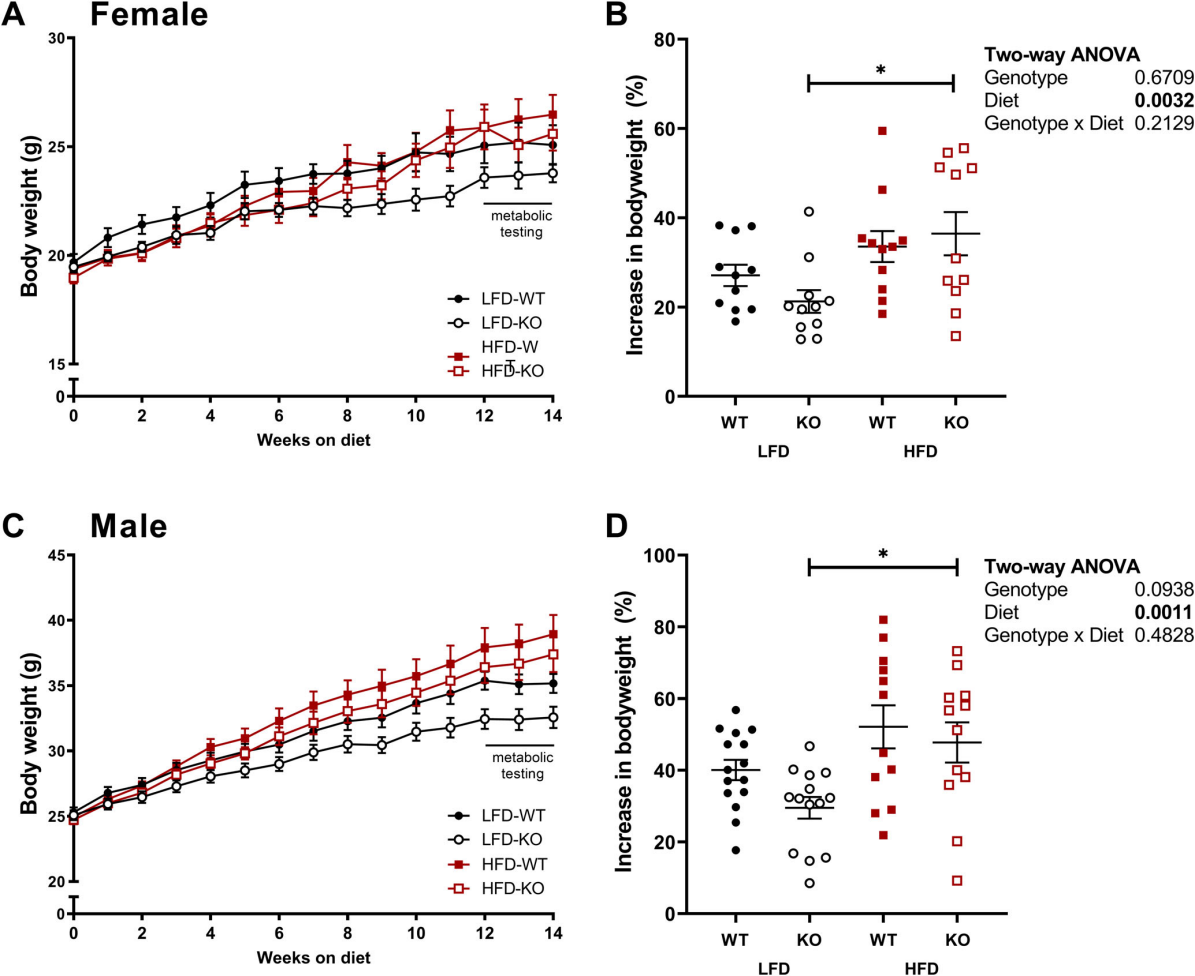
Bodyweights increase in response to HFD, particularly in KO mice. Weekly bodyweights from entry into study through 14 weeks of dietary intervention in female and male mice (*A*, *C*). Percentage increase in bodyweight between entry into study and 12 weeks of dietary intervention (*B*, *D*) were compared using two‐way ANOVA with genotype and diet as factors. Significant difference of diet within genotypes (Šidák's post hoc test) are indicated in (*B*) and (*D*), *p < 0.05. Group numbers: female LFD‐WT, *n* = 11; female LFD‐KO, *n* = 11; female HFD‐WT, *n* = 11; female HFD‐KO, *n* = 11; male LFD‐WT, *n* = 15; male LFD‐KO, *n* = 14; male HFD‐WT, *n* = 12; male HFD‐KO, *n* = 12.

To assess whether preptin deficiency alters the response of mice to a HFD, we fed WT and preptin KO mice either HFD or LFD for 14 weeks as indicated in Fig. 1. The bodyweight and body composition of WT and KO mice of both sexes were similar before starting the dietary intervention (Fig. 2*A*, *C*; Table S2). All mice gained weight during the dietary intervention period (between 9.2% and 84.5%; Fig. 2), and two‐way ANOVA showed diet significantly increased weight gain in both male and female mice. Post hoc testing showed that differences in weight gain between diets were only significant in KO mice in both males and females (Fig. 2*B*, *D*).

Lean mass was not affected by a HFD in either sex (Table 1). Absolute and relative fat mass were increased in female mice fed a HFD compared to those fed a LFD (Table 1). Post hoc tests indicated the body fat difference in diets was significant for female KO but not WT mice (Table 1). Similarly, fat mass was increased from 27% to 36% in male mice of either genotype fed a HFD compared to those fed a LFD; however, there was no effect of genotype on fat mass in male mice (Table 1). HFD‐fed mice had a lower BMD than LFD‐fed mice with no effect of genotype (Table 1).

**Table 1:** Body Composition Following Dietary Intervention Shows Increased Fat Mass in Response to HFD

	LFD			HFD			Two‐way ANOVA		
	WT	KO	Difference	WT	KO	Difference	Genotype	Diet	Genotype × diet
Female	*n* = 11	*n* = 11		*n* = 11	*n* = 11				
Lean mass (g)	17.2 ± 0.5	16.9 ± 0.2	−0.3 ± 0.5	17.7 ± 0.4	17.1 ± 0.4	−0.6 ± 0.6	0.29	0.30	0.70
Fat mass (g)^c^	5.6 ± 0.6	4.5 ± 0.2	−1.2 ± 0.6	6.9 ± 0.6	6.4 ± 0.4	−0.5 ± 0.7	0.08	0.0016	0.46
Fat mass (%)^c^	24.2 ± 1.4	20.8 ± 0.6	−3.4 ± 1.5	27.6 ± 1.6	26.9 ± 1.1	−0.7 ± 1.9	0.10	0.0004	0.27
BMD (g/cm^2^)^b^, ^c^	0.0607 ± 0.0013	0.0621 ± 0.0010	0.0014 ± 0.0017	0.0566 ± 0.0005	0.0570 ± 0.0005	−0.0005 ± 0.0007	0.29	<0.0001	0.60
Male	*n* = 13	*n* = 9		*n* = 12	*n* = 9				
Lean mass (g)	23.6 ± 0.5	22.2 ± 0.4	−1.3 ± 0.7	23.6 ± 0.4	23.3 ± 0.5	−0.3 ± 0.7	0.10	0.27	0.31
Fat mass (g)^b^ ^,^ ^c^	9.2 ± 0.5	7.9 ± 0.5	−1.4 ± 0.7	14.0 ± 1.6	13.2 ± 1.3	−0.7 ± 2.1	0.28	<0.0001	0.75
Fat mass (%)^b^, ^c^	28.0 ± 1.2	25.9 ± 1.2	−2.1 ± 1.7	36.1 ± 3.0	35.6 ± 2.3	−0.5 ± 3.8	0.50	<0.0001	0.67
BMD (g/cm^2^)^b^, ^c^	0.0608 ± 0.0006	0.0597 ± 0.0008	−0.0011 ± 0.0010	0.0557 ± 0.0004	0.0562 ± 0.0007	0.0005 ± 0.0008	0.68	<0.0001	0.27

^a^ Genotype effect within the same diet.

^b^ Dietary effect within WT mice.

^c^ Dietary effect within KO mice (*p* < 0.05, Šidák's post hoc test). Data are means ± SEM.

Regression analysis of the entire cohort revealed significantly lower postdietary intervention body mass in KO compared to WT mice (−1.5 ± 0.7 g, *p* = 0.03) when corrected for sex, diet, and prediet body mass (adjusted *R* ^2^ = 0.78, F(4,82) = 77.11, *p* < 0.001). Post‐diet lean mass was also slightly but significantly lower in KO mice (−0.6 ± 0.27 g, *p* = 0.03) when corrected for sex, diet, and prediet lean mass (adjusted *R* ^2^ = 0.85, F(4,82) = 124.7, *p* < 0.001). Postdiet fat mass was not affected by genotype when corrected for sex, diet, and prediet mass.

### Blood glucose is reduced in male KO mice on a LFD but not HFD, and dietary impact on glucose‐stimulated insulin secretion is blunted in KO female mice

**Fig. 3:**
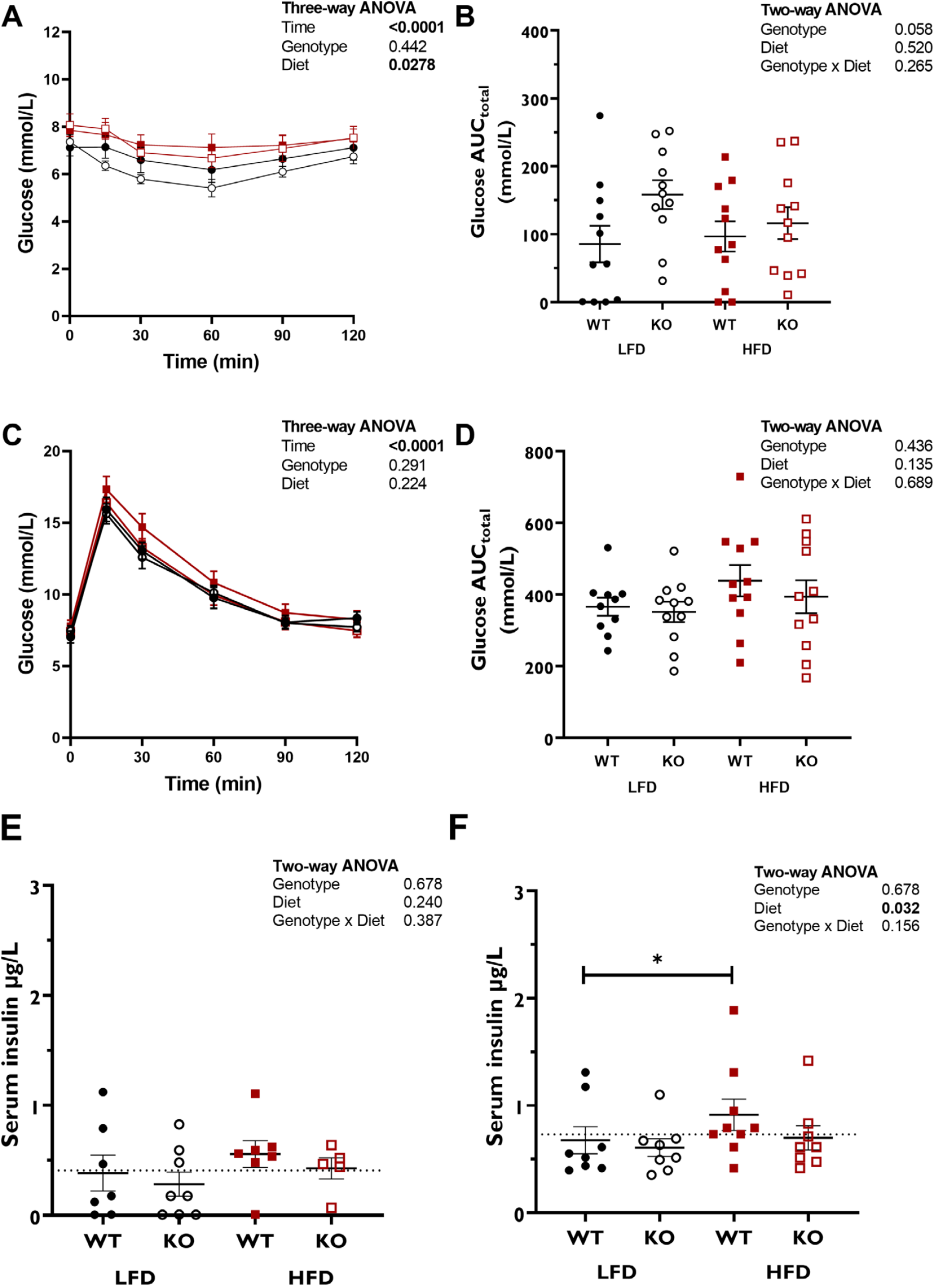
Glucoses response to ITT (*A, B*) and GTT (*C*–*F*) are not altered by preptin KO in female mice. For mean glucose concentrations (*A*, *C*), data were compared using three‐way ANOVA with genotype, diet, and time as factors. ANOVA *p* values are shown for factors, with only significant interactions shown. Glucose area under the curve for ITT (*B*) and for GTT (*D*); data were compared using two‐way ANOVA. Serum insulin concentrations at start (*E*) and 30‐min (*F*) time point of GTT were compared using two‐way ANOVA. Dotted line indicates overall mean serum insulin. Post hoc testing was Šidák's multiple comparisons assessing diet effect within each genotype. Group numbers: LFD‐WT, *n* = 11; LFD‐KO, *n* = 11; HFD‐WT, *n* = 11; HFD‐KO, *n* = 11. *p < 0.05.

Female HFD‐fed mice had slightly lower nonfasted glucose at the start of the intervention than LFD‐fed mice (*p* = 0.0496; Table 2). After 12 weeks of dietary intervention there were no significant differences in nonfasted blood glucose between groups. Despite the absence of dietary differences in fasted glucose in females, female mice fed a HFD generally had higher blood glucose concentrations than female mice fed a LFD during an ITT, but there were no genotype differences (*p* = 0.0278; Fig. 3*A*). There were no significant differences in the magnitude of insulin‐induced glucose lowering (AUC) between any of the groups in female mice, although genotype effect was *p* = 0.058, possibly reflecting the slightly stronger overall response to insulin in the female KO mice on LFD (Fig. 3*B*). Female mice displayed no significant differences in the blood glucose response to an oral GTT after 13 weeks of dietary intervention (Fig. 3*C*, *D*). However, HFD significantly increased 30‐min serum insulin levels during the GTT, and post hoc testing revealed this was only in the WT mice (*p* = 0.032; Fig. 3*E*, *F*). Neither genotype nor diet impacted HOMA‐IR or HOMA‐β as calculated from fasting insulin and glucose levels at the start of the GTT (Figure S1).

**Table 2:** Blood Glucose Is Lower in Male Preptin KO Mice

	LFD			HFD			Two‐way ANOVA		
	WT	KO	Difference	WT	KO	Difference	Genotype	Diet	Genotype × diet
Female	*n* = 9–11	*n* = 10–11		*n* = 10–11	*n* = 8–11				
Nonfasted glucose, 0–2 weeks (mmol/L)	8.9 ± 0.1	8.7 ± 0.2	−0.2 ± 0.3	8.6 ± 0.2	8.2 ± 0.1	−0.5 ± 0.3	0.09	0.0496	0.47
Nonfasted glucose, 10–12 weeks (mmol/L)	8.3 ± 0.3	8.2 ± 0.1	−0.1 ± 0.3	8.5 ± 0.2	8.0 ± 0.2	−0.5 ± 0.3	0.25	0.99	0.37
Fasted glucose (mmol/L)	7.0 ± 0.4	7.5 ± 0.3	0.4 ± 0.6	7.8 ± 0.4	7.4 ± 0.4	−0.4 ± 0.6	0.92	0.39	0.33
Nonfasted serum NEFA (mmol/L)	0.55 ± 0.04	0.56 ± 0.05	0.01 ± 0.07	0.56 ± 0.05	0.56 ± 0.05	0.00 ± 0.08	0.93	0.92	0.94
Nonfasted serum triglycerides (mmol/L)	0.99 ± 0.07	1.09 ± 0.06	0.09 ± 0.09	0.98 ± 0.08	0.87 ± 0.06	−0.11 ± 0.10	0.90	0.11	0.14
Nonfasted liver triglycerides (μg/g protein)^c^	16.1 ± 1.9	11.3 ± 1.2	−4.8 ± 3.2	18.0 ± 2.1	24.7 ± 4.2	6.7 ± 3.5	0.69	0.003	0.02
Male	*n* = 13–15	*n* = 12–13		*n* = 10–11	*n* = 10–11				
Nonfasted glucose, 0–2 weeks (mmol/L)	10.2 ± 0.3	9.4 ± 0.2	−0.8 ± 0.4	9.7 ± 0.4	9.0 ± 0.24	−0.7 ± 0.4	0.01	0.12	0.86
Nonfasted glucose, 10–12 weeks (mmol/L)	10.0 ± 0.3	9.1 ± 0.3	−0.9 ± 0.4^a^	9.8 ± 0.3	9.1 ± 0.2	−0.7 ± 0.4	0.007	0.69	0.78
Fasted glucose (mmol/L)^c^	9.2 ± 0.4	7.5 ± 0.4	−1.6 ± 0.5^a^	9.6 ± 0.7	9.7 ± 0.6	0.1 ± 0.9	0.15	0.02	0.09
Nonfasted serum NEFA (mmol/L)	0.65 ± 0.05	0.64 ± 0.04	−0.01 ± 0.07	0.57 ± 0.05	0.70 ± 0.08	0.13 ± 0.1	0.25	0.80	0.42
Nonfasted serum triglycerides (mmol/L)^b^	1.93 ± 0.14	1.45 ± 0.11	−0.48 ± 0.17^a^	1.50 ± 0.13	1.56 ± 0.10	0.06 ± 0.19	0.10	0.21	0.04
Nonfasted liver triglycerides (μg/g protein)	16.7 ± 1.9	17.2 ± 1.5	0.5 ± 3.0	23.7 ± 3.2	18.9 ± 1.8	−4.8 ± 3.1	0.34	0.05	0.24

^a^ Genotype effect within the same diet.

^b^ Dietary effect within WT mice.

^c^ Dietary effect within KO mice (*p* < 0.05, Šidák's post hoc test). Fasted glucose values are from the oral GTT. Data are means ± SEM.

**Fig. 4:**
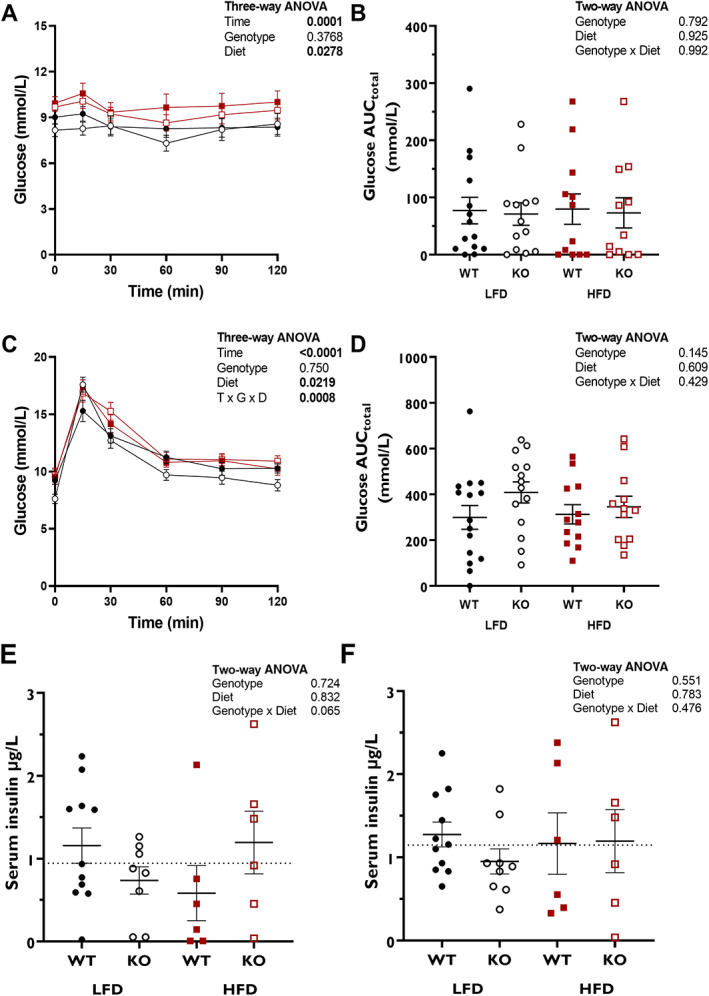
Glucose responses to ITT (*A*, *B*) and GTT (*C*–*F*) are not altered by preptin KO in male mice. For mean glucose concentrations (*A*, *C*), data were compared using three‐way ANOVA with genotype, diet, and time as factors. ANOVA *p* values are shown for factors, with only significant interactions shown. Glucose area under the curve for ITT (*B*) and for GTT (*D*); data were compared using two‐way ANOVA. Serum insulin concentrations at start (*E*) and 30‐min (*F*) time point of GTT were compared using two‐way ANOVA. Dotted line indicates overall mean serum insulin. Group numbers: LFD‐WT, *n* = 15; LFD‐KO, *n* = 14; HFD‐WT, *n* = 12; HFD‐KO, *n* = 12.

Male KO mice had lower nonfasted blood glucose concentrations than WT mice at the start of the study (*p* = 0.01; Table 2). Later in the dietary intervention, male KO mice on LFD had reduced fasting and nonfasting blood glucose compared to WT. HFD‐fed male mice had higher fasted glucose at the end of the study period (Table 2). HFD‐fed males had higher blood glucose concentrations over the course of the ITT than LFD‐fed male mice (Fig. 4*A*), but, as with the females, there were no differences in the glucose response to insulin (Fig. 4*B*). Diet also had an overall impact on the response to a GTT (Fig. 4*C*), but there were no differences in AUC (Fig. 4*D*), suggesting no difference in the deviation of blood glucose in response to a bolus between groups. This was accompanied by no differences in serum insulin levels, HOMA‐IR, or HOMA‐β (Fig. 4*E*, *F*, Fig. S1).

### Nonesterified fatty acids and triglyceride concentrations

Serum NEFA and triglyceride concentrations were similar between genotypes and dietary groups in both sexes of mice after 14 weeks of dietary intervention (Table 2). Serum triglycerides were decreased in male HFD‐fed WT mice compared to LFD‐fed WT mice (*p* = 0.03; Table 2), whereas an opposite, albeit nonsignificant, effect was seen in the KO mice, leading to an interaction effect (*p* = 0.04). The HFD led to increased liver triglycerides in both sexes, but the effect failed to reach statistical significance for males (*p* = 0.052; Table 2). In females, this was driven by an increase in KO mice (*p* < 0.0001), with no significant increase in WT, while in males, the largest difference was seen in the WT group, although this did not reach statistical significance.

### Preptin KO affects bone microarchitecture only on LFD in males

**Fig. 5:**
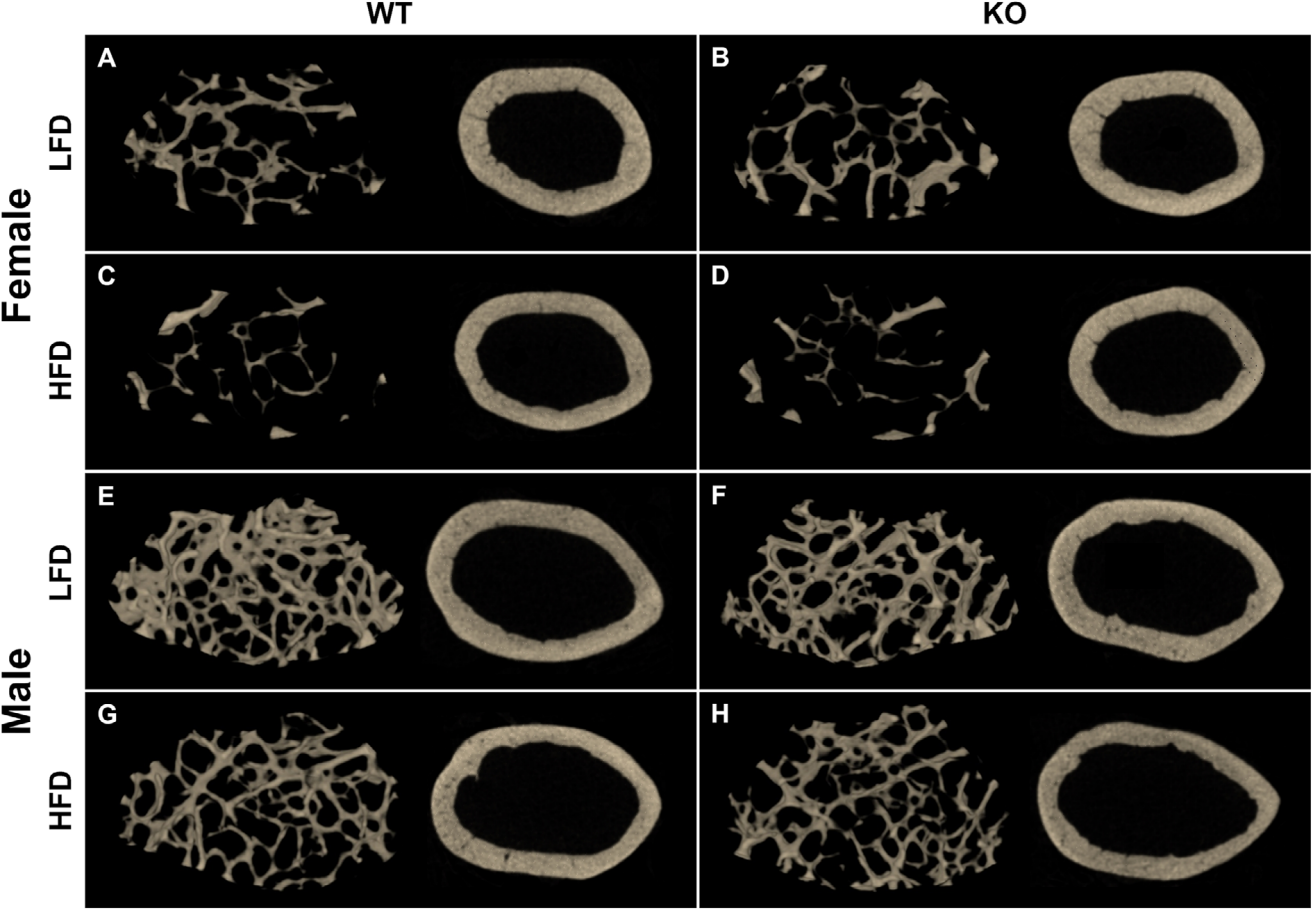
MCT reconstructions indicate bone loss in response to HFD. MCT reconstructions of trabecular and cortical bone in distal femur in representative female (*A*–*D*) and male (*E*–*H*) mice after 14 weeks of a LFD or HFD.

In female mice, HFD led to reduced trabecular bone, including a 49% decrease in bone volume fraction, decreased trabecular number and thickness, compared to LFD‐fed female mice, with no effect of genotype (Table 3; Fig. 5). Cross‐sectional cortical tissue area and bone area and cortical thickness were lower in HFD‐fed than LFD‐fed female mice, with no effect of genotype (Table 3; Fig. 5). Neither a HFD nor preptin KO affected tissue mineral density in females (Table 3).

**Table 3:** Microarchitectural Parameters of Distal Femur in Female Mice Indicate Bone Loss in Response to HFD

	LFD			HFD			Two‐way ANOVA		
	WT (*n* = 11)	KO (*n* = 11)	Difference	WT (*n* = 11)	KO (*n* = 11)	Difference	Genotype	Diet	Genotype × diet
Femur length (mm)	15.7 ± 0.2	15.5 ± 0.2	−1.2 ± 0.2	15.3 ± 0.2	15.5 ± 0.1	0.2 ± 0.2	0.70	0.17	0.23
Trabecular bone									
Bone volume fraction (%)^b^ ^,^ ^c^	8.3 ± 0.7	7.6 ± 0.8	−0.7 ± 1.1	4.0 ± 0.4	4.2 ± 0.4	0.2 ± 0.6	0.68	<0.001	0.42
Trabecular thickness (μm)^b^ ^,^ ^c^	53.7 ± 1.7	53.7 ± 1.6	0.1 ± 2.3	45.3 ± 1.1	47.5 ± 0.9	2.2 ± 1.5	0.41	<0.001	0.44
Trabecular separation (μm)^b^ ^,^ ^c^	286 ± 6	305 ± 10	19 ± 12	334 ± 9	330 ± 6	−4 ± 11	0.35	<0.001	0.17
Trabecular number (1/mm)^b^ ^,^ ^c^	1.53 ± 0.09	1.38 ± 0.11	−0.15 ± 0.14	0.87 ± 0.08	0.87 ± 0.06	0.01 ± 0.1	0.44	<0.001	0.40
Cortical bone									
Cross‐sectional tissue area (mm^2^)^b^ ^,^ ^c^	1.86 ± 0.03	1.86 ± 0.02	0.00 ± 0.04	1.76 ± 0.02	1.78 ± 0.02	0.02 ± 0.03	0.73	0.001	0.64
Cross‐sectional bone area (mm^2^)^b^ ^,^ ^c^	0.98 ± 0.03	1.00 ± 0.02	0.02 ± 0.04	0.89 ± 0.02	0.90 ± 0.01	0.01 ± 0.02	0.55	<0.001	0.74
Medullary area (mm^2^)	0.88 ± 0.01	0.86 ± 0.02	−0.02 ± 0.02	0.87 ± 0.01	0.88 ± 0.01	0.02 ± 0.02	0.79	0.82	0.14
Cortical thickness (mm)^b^ ^,^ ^c^	0.2160 ± 0.0055	0.2213 ± 0.0038	0.0053 ± 0.0067	0.2016 ± 0.0022	0.2022 ± 0.0025	0.0006 ± 0.0034	0.43	<0.001	0.54
Tissue mineral density (g/cm^3^)	1.31 ± 0.01	1.31 ± 0.01	0.00 ± 0.01	1.31 ± 0.01	1.31 ± 0.1	0.01 ± 0.02	0.70	0.80	0.76
Polar moment of inertia (mm^4^)^c^	0.45 ± 0.02	0.45 ± 0.01	0.00 ± 0.02	0.39 ± 0.01	0.39 ± 0.01	0.00 ± 0.02	0.82	<0.001	0.97

^a^ Genotype effect within the same diet.

^b^ Dietary effect within WT mice.

^c^ Dietary effect within KO mice (*p* < 0.05, Šidák's post hoc test). Data are means ± SEM.

In male mice, femur lengths were shorter in male HFD‐fed WT mice than in LFD‐fed WT mice (*p* = 0.003; Table 4). The HFD also negatively affected trabecular bone in males, as illustrated by the 35% reduction in bone volume fraction compared to LFD‐fed mice (Table 4; Fig. 5). In LFD‐fed males, bone volume fraction was 17% lower in KO than in WT mice driven by reduced trabecular number. There was no effect of genotype in HFD‐fed male mice (Table 4; Fig. 5). Similar to the phenotype displayed in female mice, cross‐sectional cortical tissue area and bone area were lower in HFD‐fed than LFD‐fed male mice (Table 4; Fig. 5). Male HFD‐fed mice also demonstrated 8.6% lower cortical thickness than LFD‐fed mice (Table 4; Fig. 5). LFD‐fed KO mice had 4.6% thinner cortical bone than LFD‐fed WT mice (*p* = 0.04). In male mice only, tissue mineral density demonstrated a genotype × diet interaction effect (*p* = 0.02), where LFD‐fed KO mice tended to have elevated total mineral density compared to LFD‐fed WT mice (*p* = 0.10), and HFD‐fed KO mice tended to have decreased TMD compared to HFD‐fed WT mice (Table 4). To summarize, HFD consistently led to a deterioration of both trabecular and cortical parameters. Preptin KO did not affect bone microarchitecture in females, but in males, preptin KO mice had reduced trabecular and cortical bone on the LFD only.

**Table 4:** Microarchitectural Parameters of Distal Femur in Male Mice Indicate Bone Loss in Response to HFD

	LFD			HFD			Two‐way ANOVA		
	WT (*n* = 15)	KO (*n* = 14)	Difference	WT (*n* = 12)	KO (*n* = 12)	Difference	Genotype	Diet	Genotype × diet
Femur length (mm)^b^	15.6 ± 0.1	15.6 ± 0.1	0.1 ± 0.1	15.1 ± 0.2	15.4 ± 0.1	0.3 ± 0.2	0.29	0.007	0.14
Trabecular bone									
Bone volume fraction (%)^b^	16.5 ± 0.6	13.7 ± 0.9	−2.8 ± 1.1^a^	9.6 ± 0.4	9.8 ± 0.5	0.1 ± 0.6	0.13	<0.001	0.09
Trabecular thickness (μm)^b^ ^,^ ^c^	53.3 ± 0.9	51.7 ± 1.4	−1.9 ± 1.6	46.4 ± 1.1	45.9 ± 1.0	− 0.5 ± 1.4	0.28	<0.001	0.53
Trabecular separation (μm)^b^	199 ± 2	212 ± 6	8 ± 7	226 ± 4	228 ± 8	2 ± 9	0.41	0.002	0.59
Trabecular number (1/mm)^b^ ^,^ ^c^	3.08 ± 0.08	2.63 ± 0.15	−0.34 ± 0.18^a^	2.08 ± 0.07	2.14 ± 0.12	0.06 ± 0.13	0.24	<0.001	0.10
Cortical bone									
Cross‐sectional tissue area (mm^2^)^b^	2.25 ± 0.04	2.17 ± 0.05	−0.08 ± 0.06	2.10 ± 0.04	2.12 ± 0.06	0.02 ± 0.07	0.55	0.03	0.33
Cross‐sectional bone area (mm^2^)^b^ ^,^ ^c^	1.09 ± 0.02	1.02 ± 0.03	−0.07 ± 0.04^a^	0.95 ± 0.01	0.95 ± 0.02	0.00 ± 0.03	0.15	<0.001	0.13
Medullary area (mm^2^)	1.16 ± 0.03	1.16 ± 0.03	0.01 ± 0.04	1.15 ± 0.03	1.17 ± 0.04	0.02 ± 0.05	0.87	0.97	0.72
Cortical thickness (mm)^b^ ^,^ ^c^	0.2096 ± 0.0034	0.2001 ± 0.0045	−0.0099 ± 0.0056^a^	0.1876 ± 0.0026	0.1869 ± 0.0018	−0.0007 ± 0.0031	0.14	<0.001	0.20
Tissue mineral density (g/cm^3^)^b^	1.29 ± 0.01	1.31 ± 0.01	0.02 ± 0.01	1.31 ± 0.01	1.29 ± 0.01	−0.02 ± 0.01	0.97	0.60	0.02
Polar moment of inertia (mm^4^)^b^	0.63 ± 0.02	0.57 ± 0.02	−0.06 ± 0.03	0.52 ± 0.02	0.53 ± 0.03	0.01 ± 0.03	0.32	0.002	0.16

^a^ Genotype effect within the same diet.

^b^ Dietary effect within WT mice.

^c^ Dietary effect within KO mice (*p* < 0.05, Šidák's post hoc test). Data are means ± SEM.

### Bone strength is reduced by HFD in WT mice

Fracture load was 21% lower in HFD‐fed female mice than in LFD‐fed female mice (*p* = 0.02; Table 5), with this effect stronger in WT than KO mice (*p* = 0.005 and *p* = 0.57, respectively). Similarly, in male mice, fracture load was 11% lower in the HFD group than in LFD‐fed males (*p* = 0.009; Table 5), with this effect stronger in WT than KO mice (*p* = 0.006 and *p* = 0.33, respectively). Fracture load was also decreased in LFD‐fed KO males compared to LFD‐fed WT males (*p* = 0.04). Displacement to fracture load was not affected by diet or genotype in either sex (Table 5). Mice of both sexes demonstrated decreased stiffness in HFD‐fed mice compared to LFD‐fed mice (27% lower in females, 22% lower in males, Table 5). This effect was stronger in WT than in KO mice.

**Table 5:** Biomechanical Testing of Femurs Indicates Impaired Strength with HFD

	LFD			HFD			Two‐way ANOVA		
	WT	KO	Difference	WT	KO	Difference	Genotype	Diet	Genotype × diet
Female	*n* = 11	*n* = 8		*n* = 11	*n* = 8				
Fracture load (N)^a^	18.0 ± 1.5	15.6 ± 0.7	−2.4 ± 1.9	13.5 ± 0.7	13.5 ± 0.5	0.1 ± 0.9	0.58	0.02	0.13
Displacement to fracture load (mm)	0.43 ± 0.02	0.41 ± 0.01	−0.02 ± 0.03	0.38 ± 0.2	0.40 ± 0.03	0.02 ± 0.03	1.00	0.25	0.29
Stiffness (N/mm)^b^	420 ± 37	392 ± 21	−28 ± 47	295 ± 20	307 ± 28	13 ± 33	0.94	0.003	0.31
Male	n = 14	n = 14		n = 11	n = 10				
Fracture load (N)^b^	17.3 ± 0.5	15.0 ± 0.8	−2.3 ± 1.0^a^	13.1 ± 0.4	13.8 ± 0.9	0.7 ± 1.0	0.13	0.009	0.21
Displacement to fracture load (mm)	0.37 ± 0.01	0.35 ± 0.01	−0.02 ± 0.02	0.34 ± 0.01	0.34 ± 0.02	0.00 ± 0.02	0.24	0.15	0.74
Stiffness (N/mm)^b^	621 ± 44	508 ± 45	−114 ± 63	411 ± 30	461 ± 53	51 ± 59	0.38	0.009	0.10

^a^ Genotype effect within the same diet.

^b^ Dietary effect within WT mice.

^c^ Dietary effect within KO mice (*p* < 0.05, Šidák's post hoc test). Data are means ± SEM.

## Discussion

Preptin has been proposed as a potential therapeutic target to improve metabolic dysfunction in individuals with T2DM; however, whether preptin impacted metabolic dysfunction and bone loss associated with a HFD was unknown. Therefore, we measured the effects of both HFD and LFD over a 14‐week period in the novel preptin KO mouse, focusing on metabolism and skeletal phenotype. The sexually dimorphic effect of HFD on body composition and glucose homeostasis in mice is a well‐known phenomenon that is becoming increasingly understood.^(^ ^43^, ^44^, ^45^, ^46^ ^)^ In addition, we have already noted sexually dimorphic phenotypes in preptin KO mice.^(^ ^34^ ^)^ We therefore examined our male and female mice separately.

We found that this HFD intervention increased bodyweight and fat mass and increased liver triglycerides and lowered BMD in mice of both sexes relative to the LFD. There was only a modest effect on fasted blood glucose in males. The short duration and moderate fat content of our HFD intervention might explain the modest dietary impact on glucose homeostasis; however, there was a significant effect on fat mass gain, liver triglycerides (particularly in females), and BMD in both males and females, indicating that metabolic dysfunction was occurring. In addition, 30‐min insulin levels were elevated in HFD‐fed female mice, suggesting an attempt to adapt to metabolic dysfunction that was absent in the KO females. This fits with our previous data showing that preptin ablation in female mice led to diminished GSIS.^(^ ^34^ ^)^

In this study, nonfasted blood glucose concentrations were lower in male KO mice than in WT mice at the start and end of the dietary intervention period in the LFD study arm despite the absence of major differences in weight and fat mass. This suggests that preptin deficiency may improve overall physiological glucose regulation in a nonstressed state in males specifically. However, we previously demonstrated no differences in weekly nonfasted blood glucose measures between genotypes in male mice fed a standard laboratory chow diet,^(^ ^34^ ^)^ so this will need further exploration.

In general, we found preptin KO mice to be slightly lighter under LFD but not HFD conditions, and the difference between diets was significant only in KO mice in both sexes. Our previous study on these mice showed that under standard feed and housing conditions, both male and female KO mice tended to be lighter once their growth rate slowed down in adulthood and up to 48 weeks of age, although this did not reach statistical significance in either sex.^(^ ^34^ ^)^ Notably we found no difference in organ weights in our previous study that would explain this change, so preptin deficiency may have a subtle impact on weight gain in adult mice, but the mechanism is unclear. We also detected no impact of KO on *Igf2* gene expression levels that might explain size difference.

In addition to a sexually dimorphic impact on β‐cell function, female preptin KO mice also had significantly higher fat mass differences between diets and had higher liver triglyceride levels in HFD relative to LFD. Excess liver triglyceride levels in HFD females lacking preptin suggest preptin may be beneficial for limiting fat storage in the liver under HFD conditions. Interestingly, our data suggest a differential impact of preptin deficiency on liver triglyceride storage between males and females, so elevated preptin in diabetic states could be more beneficial to females than males regarding triglyceride accumulation in the liver, which is a hallmark of nonalcoholic fatty liver disease.^(^ ^47^ ^)^ The gene expression of *Igf2* (exon 2–3) in liver of preptin KO mice was previously assessed and found not to be different between KO and WT mice. However, an impact of differential processing of mature IGF‐II protein cannot be excluded, and further analysis of this phenomenon might uncover sexually dimorphic responses of liver to dietary fat.

Multiple observational human studies have linked elevated circulating preptin to metabolic dysfunction, including reports of elevated concentrations in obesity,^(^ ^14^, ^17^ ^)^ T2DM,^(^ ^12^ ^)^ gestational diabetes mellitus,^(^ ^15^ ^)^ and polycystic ovarian syndrome.^(^ ^13^, ^18^, ^19^ ^)^ Direct comparisons between observational human studies are complicated, as each study uses a different ELISA or radioimmunoassay protocol. Nevertheless, given the number of studies reporting similar results, if preptin had a causal role in metabolic dysfunction, we would expect to see some effect of the ablation of preptin on metabolic function under metabolic stress conditions. As preptin is secreted alongside insulin from β‐cells, increases in circulating preptin concentrations seen in observational studies might have resulted from increased insulin secretion, as opposed to its expression being explicitly increased and having a causative role in the development of metabolic dysfunction. Our data support the idea that preptin is an insulin secretagogue, particularly in female mice. The presence of lower blood glucose levels in KO males on a LFD may indicate that this putative insulin secretagogue has other effects on glucose management that could drive elevated blood glucose, although perhaps only evident under LFD conditions where we would not expect to see dysfunction in insulin secretory capacity. Our data indicate this may be male‐specific. Our model is a global ablation model, specifically because it has not been excluded that preptin is expressed in tissues other than the islet β‐cells, where it may impact insulin action rather than just secretion. Little evidence of this has been presented to date, other than in a report of a rare case of monogenic diabetes resulting from an IGF2 gene disruption, which resulted in not only short adult stature, as expected, but also extreme hepatic insulin resistance.^(^ ^48^ ^)^

As expected, our moderate HFD had impacts on bone structure. We found that mice fed a HFD had decreased trabecular bone and cortical thickness compared to mice fed a LFD. While we did not further explore the cellular mechanisms underlying the bone loss in the current study, this occurrence has been well documented in both female and male rodents^(^ ^49^, ^50^, ^51^, ^52^, ^53^ ^)^ and occurs via increased bone resorption^(^ ^54^ ^)^ and decreased bone formation.^(^ ^55^ ^)^ The HFD in our study consisted of 46% calories from fat, which is lower than the 60% fat diets used in many studies.^(^ ^54^, ^56^ ^)^ Notably, bone microarchitecture and strength in these mice deteriorated markedly, even in the absence of severe obesity or overt metabolic dysfunction, consistent with other studies showing rapid bone loss with a HFD.^(^ ^54^ ^)^ Unlike the metabolic phenotypes, the magnitude of effects on bone microarchitecture was similar between male and female mice. Given that previous in vivo and ex vivo experiments demonstrated that exogenous preptin increased bone formation via effects on osteoblasts,^(^ ^31^, ^32^, ^33^ ^)^ we hypothesized that the total ablation of preptin expression would lead to lower levels of bone in our KO mouse when fed a HFD. Preptin deficiency did not affect bone microarchitecture or strength in mice of either sex fed a HFD. However, in male mice fed a LFD, the trabecular bone volume fraction, cortical thickness, and load required to fracture the femur were lower in KO mice than in WT mice. The mechanisms that led to the appearance of this phenotype in LFD‐fed mice only remain unclear, although notably, the LFD‐fed KO mice also tended to have lower bodyweight than WT controls. Therefore, further exploration of the mechanisms that led to the bone phenotype in LFD‐fed mice will be of interest. The LFD had slightly lower fat (14%) than our standard laboratory chow (18%), and since weight gain continued following the dietary transition, there were no clear negative effects of the LFD. Observational studies suggest a positive relationship between serum preptin concentrations and BMD.^(^ ^57^, ^58^ ^)^ Preptin levels were also decreased after menopause^(^ ^57^ ^)^ and in ovariectomized rats.^(^ ^59^ ^)^ In contrast, we have been unable to detect preptin in adult mouse serum.^(^ ^34^ ^)^ Nevertheless, it would be of interest to investigate whether preptin is affected by feeding mice a HFD and whether preptin concentrations are related to any of the bone parameters measured in our study.

It is possible we did not observe an overt effect of preptin deficiency on glucose or skeletal homeostasis under HFD conditions as preptin is only one physiological variable in a network of feedback loops that regulate these systems.^(^ ^60^, ^61^, ^62^ ^)^ Thus, most effects of preptin KO that we observed occurred in mice fed a LFD. Preptin may play a larger role in metabolic and skeletal health under healthy conditions, while in the context of metabolic dysfunction, other physiological variables could exert a stronger influence.

In conclusion, we showed that feeding mice a HFD for 14 weeks negatively affected liver triglycerides and bone microarchitecture in mice, and preptin removal worsened the response to metabolic stress in females but did not affect the male response to a HFD. Further studies are required to determine whether preptin deficiency leads to an altered skeletal phenotype in mice fed a standard diet with advancing age.

## Author Contributions

**Emma J Buckels:** Formal analysis; funding acquisition; investigation; supervision; writing – original draft; writing – review and editing. **Joey Tan:** Data curation; formal analysis; investigation; writing – review and editing. **Huai‐L Hsu:** Data curation; investigation; writing – review and editing. **Yuting Zhu:** Methodology; resources. **Christina M Buchanan:** Conceptualization; funding acquisition; resources; writing – review and editing. **Brya G Matthews:** Conceptualization; funding acquisition; investigation; project administration; resources; supervision; writing – review and editing. **Kate L Lee:** Conceptualization; data curation; formal analysis; funding acquisition; investigation; project administration; resources; supervision; writing – review and editing.

## Funding Information

This work was funded by a University of Auckland Faculty Research Development grant to Dr. Buchanan; a Health Research Council of New Zealand Emerging Researcher First Grant to Dr. Lee; a Health Research Council of New Zealand Sir Charles Hercus Fellowship to Dr. Matthews; an American Society of Bone and Mineral Research Rising Star Award to Dr. Matthews; and a Maurice and Phyllis Paykel Trust project grant to Dr. Buckels.

## Disclosures

The authors have nothing to declare.

### Peer Review

The peer review history for this article is available at https://www.webofscience.com/api/gateway/wos/peer-review/10.1002/jbm4.10777.

## Supporting information


**Data S1.** Supporting Information.

## Data Availability

The data that support the findings of this study are available from the corresponding author upon reasonable request.
