# Supplementary material for: Preptin Deficiency Does Not Protect against High‐Fat Diet‐Induced Metabolic Dysfunction or Bone Loss in Mice
Source: JBMR Plus. 2023 Jun 18;7(8):e10777. doi: 10.1002/jbm4.10777 (PMC10443080; doi:10.1002/jbm4.10777)
Supplement: Supplementary file 1 — Data S1. Supporting Information. [file JBM4-7-e10777-s001.docx]

**Supplemental Figure 1: Preptin KO does not impact HOMA-IR and HOMA-β.**

Fasting glucose and insulin levels from the start of the GTT were used to calculate HOMA-IR in females (A) and males (C) and HOMA-β in females (B) and males (D). Statistics were two-way ANOVA with genotype and diet as factors. There were no significant differences and so no post-hoc tests performed.


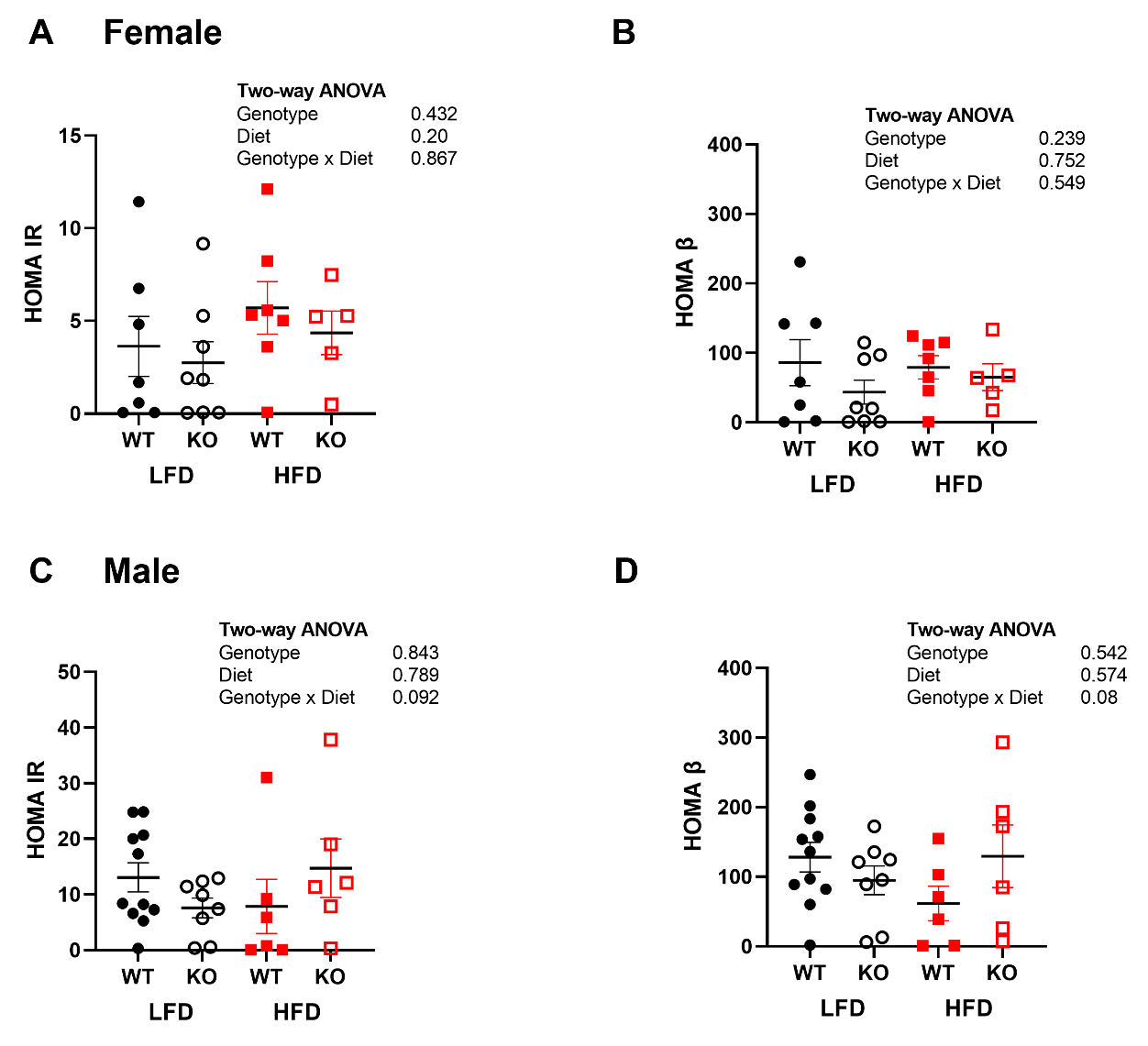


**Supplemental Table 1. Composition of** **the diets used in this study**

|  | Chow | LFD | HFD |
| --- | --- | --- | --- |
| **Macronutrient weight g per 100g of diet** |  |  |  |
| Protein | 18.6 | 18.8 | 18.4 |
| Fat | 6.2 | 6.0 | 23.5 |
| Carbohydrate | 44.2 | 59.3 | 42.3 |
| Crude Fibre | 3.5 | 5.4 | 4.7 |
| **Energy per macronutrient** |  |  |  |
| Total energy from protein (%kcal) | 24.0 | 20.7 | 20.0 |
| Total energy from fats (%kcal) | 18.0 | 14.0 | 46.0 |
| Total energy from carbohydrates (%kcal) | 58.0 | 65.3 | 34.0 |
| Energy density (kcal/g) | 3.1 | 3.7 | 4.0 |
| **Mineral weight g per 100g (selected)** |  |  |  |
| Calcium | 1.0 | 0.6 | 0.6 |
| Phosphorus | 0.7 | 0.5 | 0.4 |
| Sodium | 0.2 | 0.13 | 0.14 |
| Potassium | 0.6 | 0.8 | 0.8 |
| Magnesium | 0.2 | 0.09 | 0.1 |

.

# Supplemental Table 2: Body composition at baseline.

For each sex, data were compared using two-way ANOVA with genotype and diet as factors, followed by Tukey’s post hoc testing. † denotes a dietary effect within WT mice (p < 0.05). Data are means ± SEM. Abbreviations: wild-type (WT); knockout (KO); low-fat diet (LFD); high-fat diet (HFD).

|  | **LFD** | | | **HFD** | | | **Two-way ANOVA** | | |
| --- | --- | --- | --- | --- | --- | --- | --- | --- | --- |
|  | **WT** | **KO** | **Difference** | **WT** | **KO** | **Difference** | **Genotype** | **Diet** | **Genotype × diet** |
| **Female** | n=11 | n=11 |  | n=11 | n=11 |  |  |  |  |
| Lean mass (g) | 15.6 ± 0.3 | 15.2 ± 0.3 | -0.5 ± 0.5 | 15.0 ± 0.3 | 15.0 ± 0.2 | 0.0 ± 0.4 | 0.47 | 0.17 | 0.42 |
| Fat mass (g) | 3.1 ± 0.3 | 2.7 ± 0.2 | -0.3 ± 0.4 | 2.4 ± 0.2 | 2.5 ± 0.1 | 0.0 ± 0.2 | 0.51 | 0.09 | 0.46 |
| Fat mass (%) | 15.7 ± 1.1 | 14.8 ± 1.1 | -0.9 ± 1.4 | 14.0 ± 0.7 | 14.0 ± 0.5 | 0.0 ± 0.8 | 0.25 | 0.13 | 0.33 |
| BMD (g/cm^2^) † | 0.0477 ± 0.0006 | 0.0471 ± 0.0007 | -0.0006 ± 0.0009 | 0.0456 ± 0.0004 | 0.0469 ± 0.0006 | 0.0012 ± 0.0007 | 0.54 | 0.05 | 0.10 |
| **Male** | n=13 | n=12 |  | n=9 | n=9 |  |  |  |  |
| Lean mass (g) | 20.4 ± 0.5 | 20.2 ± 0.5 | -0.1 ± 0.7 | 20.3 ± 0.3 | 20.5 ± 0.3 | 0.2 ± 0.4 | 0.90 | 0.77 | 0.67 |
| Fat mass (g) | 3.1 ± 0.2 | 3.0 ± 0.2 | -0.1 ± 0.2 | 3.4 ± 0.2 | 3.4 ± 0.2 | 0.0 ± 0.3 | 0.78 | 0.06 | 0.97 |
| Fat mass (%) | 13.1 ± 0.4 | 13.0 ± 0.5 | -0.2 ± 0.7 | 14.5 ± 0.8 | 14.2 ± 0.7 | -0.3 ± 1.0 | 0.68 | 0.04 | 0.89 |
| BMD (g/cm^2^) | 0.0509 ± 0.0007 | 0.0506 ± 0.0008 | -0.0003 ± 0.0010 | 0.0510 ± 0.0004 | 0.0510 ± 0.0007 | 0.0000 ± 0.0008 | 0.84 | 0.71 | 0.80 |
